# Impact of Regular and Irregular Pore Distributions on the Elasticity of Porous Materials: A Microstructure-Free Finite Element Study

**DOI:** 10.3390/ma17184490

**Published:** 2024-09-13

**Authors:** Prajjayini Chakma, Yunhua Luo

**Affiliations:** Department of Mechanical Engineering, University of Manitoba, Winnipeg, MB R3T 2N2, Canada; prajjayini.chakma@umanitoba.ca

**Keywords:** porous material, pore distribution pattern, effective Young’s modulus, porosity, microstructure-free finite element modeling

## Abstract

Conventional analytical formulas for predicting the effective Young’s modulus of porous materials often rely on simplifying assumptions and do not explicitly incorporate microstructural information. This study investigates the impact of regular versus irregular pore distributions on the stiffness of porous materials using microstructure-free finite element modeling (MF-FEM). After conducting a convergence study, MF-FEM predictions were validated against experimental data and used to assess the accuracy of commonly employed analytical models. The results demonstrate that materials with irregular microstructures exhibit a rapid decrease in Young’s modulus, approaching zero at porosities slightly greater than 50%. In contrast, regular microstructures show a more gradual decline, maintaining significant stiffness until the porosity exceeds 90%. Additionally, the study reveals that some analytical formulas align better with irregular microstructures while others are more suited to regular ones, attributable to the underlying assumptions of these models. These findings underscore the necessity of considering pore distribution patterns in modeling to accurately predict the mechanical behavior of porous materials.

## 1. Introduction

Porous materials are characterized by their internal structures, including the size, shape, distribution, and connectivity of their pores. Pore size refers to the dimensions of the empty spaces within a material, ranging from microscopic to visibly large holes, while pore shape describes the configuration of these spaces, which can be spherical, elongated, or irregular. The distribution of these pores can be either uniform or random, and the connectivity can vary from interconnected to isolated. These factors collectively determine the mechanical properties of porous materials and their suitability for specific applications. For example, in biomedical engineering, bone scaffolds typically feature porosities ranging from 50 to 90%. The interconnected pores within these scaffolds allow fluids and biological cells to pass through, facilitating the gradual growth and attachment of biological tissues [1]. In the aerospace industry, companies like Gulfstream Aerospace utilize custom-designed sintered porous metal filters in turbofan air-bleed systems to ensure long-term, low-maintenance operation [2]. The size and distribution of the pores are crucial in this case, as they determine the filter’s permeability and the size of particles it can effectively capture. Other applications of porous materials include their use in batteries and supercapacitors, where their high surface area and controlled porosity enhance electrode performance by facilitating ion transport and improving energy storage efficiency. Porous materials such as aerogels are also employed as thermal insulators in the construction and aerospace industries due to their low thermal conductivity and lightweight properties. Additionally, in the automotive and building sectors, porous materials are utilized for soundproofing and noise control, effectively absorbing sound waves to reduce noise levels.

The distribution of pores within porous materials is a crucial determinant of their functional effectiveness in specific applications. Pore distributions can be categorized into regular and irregular patterns. Regular distributions feature pores that are evenly spaced and consistent in size and shape throughout the material. In an irregular pore distribution, pores vary in size, shape, and spacing, and are usually not interconnected. This kind of structure is commonly found in natural materials like wood and bones, as well as in ceramics and polymer materials, where it may form during manufacturing as an unintended byproduct. Irregular pore distributions can reduce a material’s effective mechanical properties by providing weak points for crack initiation and propagation. Chen et al. studied this type of microstructure and found that porosity was the critical microstructural parameter affecting the elastic properties of porous La_0.6_Sr_0.4_Co_0.2_Fe_0.8_O_3−δ_ (LSCF) ceramic films [3]. However, materials with random porous microstructures can still be beneficial, offering properties such as shock absorption and energy dissipation. Therefore, understanding the specific applications and the desired properties is crucial in selecting or designing materials with the appropriate pore distribution.

The properties of porous materials can typically be predicted using three main methods: experimental, analytical, and numerical approaches. Among these, the experimental method is often considered the most reliable, as it directly captures real-world scenarios and conditions. Numerous studies have been conducted experimentally to determine the mechanical properties of porous materials. One of the most commonly used manufacturing techniques is sintering. However, controlling pore distribution during the manufacturing process presents significant challenges, and experimental testing often focuses more on evaluating elasticity properties at different porosity levels rather than the specific distribution of the pores. This emphasis on porosity alone, rather than pore distribution, can limit the comprehensive understanding of a material’s performance and overall mechanical properties, which are influenced by variations in pore distribution patterns. Furthermore, experimental procedures are time-consuming and costly, leading many researchers to explore alternative methods such as analytical or numerical modeling to predict material behaviors more efficiently.

Herakovich and Baxter [4] highlighted that the study of porous media is approached differently by the mechanics and materials communities. The mechanics community often focuses on a particular pore shape, commonly spherical, and develops analytical solutions to understand mechanical properties based on pore volume fraction. For example, in the Differential Effective Medium Approach (DEMA) [5], the pore shape is assumed to be spherical, and the pores are gradually added to an original solid phase until the desired pore volume fraction is achieved. On the other hand, the materials community typically gathers experimental data on mechanical properties in relation to volume porosity and then identifies the most accurate curve that connects these properties with pore geometry or the method of manufacturing [6]. For instance, Wagh [7] developed an analytical approach that accounts for the random distribution of grain and pore sizes, incorporating an empirical constant that depends on the tortuosity of the ceramic structure. Most analytical models effectively establish the relationship between porosity and material properties, but they often do not consider pore distribution and connectivity within the material. Additionally, these analytical approaches typically rely on numerous assumptions, which can cause significant deviations from actual conditions [6,7].

In addition to analytical approaches, many numerical techniques have been developed to study porous materials. Zerhouni et al. [8] conducted a finite element study by modeling a cubic representative volume element (RVE) with both periodic and kinematically uniform boundary conditions. They focused on an isotropic microstructure featuring randomly distributed, single-sized (monodisperse) spherical voids within a uniform solid matrix, achieving a porosity level of up to 30% without any interconnections between the monodisperse pores. Moreover, the effects of both regular and irregular pore distribution patterns have not been extensively studied through experimental testing, analytical modeling, or finite element modeling in the previous literature. Therefore, it is necessary to address both regular and irregular pore distributions to investigate how the stiffness of porous materials changes with varying porosity for different microstructures.

Microstructure-free finite element modeling (MF-FEM) is an effective numerical method for investigating the elastic characteristics of materials, as it does not require generating any geometric model, making it less time-consuming and easily attainable for high volume fractions of inclusions in the matrix [9,10]. One significant advantage of MF-FEM is its ability to accommodate both regular and irregular pore distribution patterns. Its capability to handle high volume fractions and disregard specific pore geometries makes it beneficial for designing materials with controlled porosity. Additionally, while many modeling algorithms are tailored for specific pore shapes like spheres and ellipsoids, real-world porous materials often feature irregularly shaped pores, complicating the creation of accurate geometric models in traditional FEM. By avoiding the need for the explicit modeling of these particular shapes and incorporating both regular and irregular distribution patterns, MF-FEM overcomes this issue, further enhancing its utility and efficiency.

The objective of this paper is to thoroughly investigate the effect of regular versus irregular pore distributions on the elasticity of porous materials using MF-FEM, validated by experimental results. The investigation outcomes provide a basis for evaluating the applicability and accuracy of commonly used analytical formulas in predicting these properties. The layout of the paper is as follows: Section 2 presents popular analytical formulas, the MF-FEM with the creation of representative volume elements (RVE) for both irregular and regular pore distributions, and the three experimental cases used for validating MF-FEM. Section 3 presents and compares the results produced by the MF-FEM and analytical formulas with the experimental data. Section 4 discusses the applicability and accuracy of the analytical formulas in relation to the MF-FEM and experimental results. Finally, Section 5 concludes the findings of our study.

## 2. Materials and Methods

In this section, we first briefly revisit popular micromechanics models and their assumptions, including the Mori–Tanaka model, the Generalized Self-Consistent (GSC) model, the Roberts and Garboczi model, Wagh’s model, Wang’s model, and the Differential Effective Medium Approach (DEMA). Next, we describe the construction of representative volume elements (RVEs) for both irregular and regular pore distributions. Finally, we introduce three experimental cases from the literature used for validation.

### 2.1. Micromechanics Models

(1) The Mori–Tanaka Model (MT)

The Mori–Tanaka (MT) model is a well-known analytical approach for determining effective material constants. It utilizes Eshelby’s equivalent inclusion theory to determine the Eshelby tensors and applies homogenization to predict the properties of the composite [11]. This method assumes a homogeneous distribution of inclusions within a continuous matrix. It calculates the average internal stress in the matrix by transforming the strain, accounting for interactions among inclusions and the effect of a free boundary on the average elastic energy [9,12,13]. The model assumes a uniform microstructure and overlooks the impact of the size and number of inclusions. For porous materials, the values of the shear and bulk moduli for the pores are considered zero.

(1) $$G_{e}=G_{m}+\frac{f_{p}\left(G_{p}-G_{m}\right)}{1+\frac{f_{m}\left(G_{p}-G_{m}\right)}{G_{m}+\frac{G_{m}\left(9K_{m}+8G_{m}\right)}{6\left(K_{m}+2G_{m}\right)}}}$$

(2) $$K_{e}=K_{m}+\frac{f_{p}(K_{p}-K_{m})}{1+\frac{f_{m}(K_{p}-K_{m})}{K_{m}+\frac{4}{3}G_{m}}}$$

In Equations (1) and (2), $G_{e}$ represents the effective shear modulus, while $G_{m}$ and $G_{p}$ denote the shear modulus of matrix and pores, respectively. Similarly, $K_{m}$ and $K_{p}$ denote the bulk modulus of matrix and pores. The volume fractions of matrix and pores are represented by $f_{m}$ and $f_{p}$.

(2) The Generalized Self-Consistent (GSC) Model

The original two-phase self-consistent model [14], which involved directly placing a spherical inclusion into an infinite medium with unknown effective properties, has been improved to a three-phase Generalized Self-Consistent (GSC) model. This improved model incorporates three phases: a spherical inclusion, a spherical annulus composed of matrix material, and an external region consisting of an equivalent homogeneous material within an infinite medium. The expression for the effective bulk modulus is the same as that in the Mori–Tanaka model, i.e., Equation (2). The expression for the shear modulus involves three additional parameters: A, B, and C, which are given below:

(3) $$G_{e}=\left(\frac{-B+\sqrt{B^{2}-A.C}}{A}\right)\cdot G_{m}$$

(4) $$K_{e}=\left(K_{m}+\frac{f_{p}(K_{p}-K_{m})}{1+\frac{f_{m}(K_{p}-K_{m})}{K_{m}+\frac{4}{3}G_{m}}}\right)$$

$$\begin{array}{c} A=8\left(\frac{G_{p}}{G_{m}}-1\right)\left(4-5v_{m}\right)\eta_{1}f_{p}^{\frac{10}{3}}-2\left(63\left(\frac{G_{p}}{G_{m}}-1\right)\eta_{2}+2\eta_{1}\eta_{3}\right)f_{p}^{\frac{7}{3}}+252\left(\frac{G_{p}}{G_{m}}-1\right)\eta_{2}f_{p}^{\frac{5}{3}}\\ -50\left(\frac{G_{p}}{G_{m}}-1\right)\left(7-12v_{m}+8{v_{m}}^{2}\right)\eta_{2}f_{p}+4\left(7-10v_{m}\right)\eta_{2}\eta_{3} \end{array}$$

$$\begin{array}{c} B=-2\left(\frac{G_{p}}{G_{m}}-1\right)\left(1-5v_{m}\right)\eta_{1}f_{p}^{\frac{10}{3}}+2\left(63\left(\frac{G_{p}}{G_{m}}-1\right)\eta_{2}+2\eta_{1}\eta_{3}\right)f_{p}^{\frac{7}{3}}-252\left(\frac{G_{p}}{G_{m}}-1\right)\eta_{2}f_{p}^{\frac{5}{3}}\\ +75\left(\frac{G_{p}}{G_{m}}-1\right)\left(3-v_{m}\right)\eta_{2}v_{m}f_{p}+\frac{3}{2}\left(15v_{m}-7\right)\eta_{2}\eta_{3} \end{array}$$

$$\begin{array}{c} C=4\left(\frac{G_{p}}{G_{m}}-1\right)\left(5v_{m}-7\right)\eta_{1}f_{p}^{\frac{10}{3}}-2\left(63\left(\frac{G_{p}}{G_{m}}-1\right)\eta_{2}+2\eta_{1}\eta_{3}\right)f_{p}^{\frac{7}{3}}+252\left(\frac{G_{p}}{G_{m}}-1\right)\eta_{2}f_{p}^{\frac{5}{3}}\\ +25\left(\frac{G_{p}}{G_{m}}-1\right)\left(v_{m}^{2}-7\right)\eta_{2}f_{p}-\left(7+5v_{m}\right)\eta_{2}\eta_{3} \end{array}$$

The three coefficients A, B, and C are determined by the shear moduli ($G_{m},\;G_{p}$), the Poisson’s ratios ($v_{m},\;v_{p}$), and the volume fractions ($f_{m},\;f_{p}$) of the matrix and pores. The equations include three additional coefficients ($\eta_{1},\;\eta_{2}$ and $\eta_{2}$), as provided below.

$$\eta_{1}=\left(\frac{G_{p}}{G_{m}}-1\right)\left(7-10v_{m}\right)\left(7+5v_{p}\right)+105\left({v_{p}-v}_{m}\right)$$

$$\eta_{2}=\left(\frac{G_{p}}{G_{m}}-1\right)\left(7+5v_{p}\right)+35\left(1-v_{p}\right)$$

$$\eta_{2}=\left(\frac{G_{p}}{G_{m}}-1\right)\left(8-10v_{m}\right)+15\left(1-v_{m}\right)$$

(3) Roberts and Garboczi model

Roberts and Garboczi [15] developed a model to predict the elastic modulus of porous ceramics.

(5) $$\frac{E}{E_{0}}={\left(1-\frac{\phi }{\phi_{0}}\right)}^{n}$$

E and $E_{0}$ denote the effective Young’s modulus of porous material and the Young’s modulus of non-porous material, respectively. $\phi_{0}$ and n are empirical parameters that vary based on the pore microstructure. The pore shape in the model is assumed to be either spherical or ellipsoidal. For spherical pores, n=1.65 and $\phi_{0}=0.818$; for ellipsoidal pores, n=2.25 and $\phi_{0}=0.798$. The distribution of these pores is random but statistically uniform, and pore interactions are assumed to be negligible. The applicable range of porosity is between 10% and 50%.

(4) Wagh’s model:

This model was developed by Wagh et al. [7] to predict the effective Young’s modulus of porous ceramic structure dominated by randomly distributed open pores.

(6) $$E=E_{0}{\left(1-p\right)}^{m}$$

E denotes the modulus of elasticity of the porous material; $E_{0}$ is the zero-porosity elastic modulus of the ceramic material, and p is the porosity. The porosity range is considered up to 50%. The parameter m is empirical. For example, m=2.14 was used for a porosity range of 22% to 47% in the fabrication of α-alumina without sintering aid [7,16]; Phani and Mukerjee [17] found m to be 1.85 for porous epoxy resin; and Hassehnan et al. [18] demonstrated m=2 for glass with spherical pores. By following Wagh et al. [7], in this study, we considered a range of m values from 2 to 5.48.

(5) Wang’s model:

Wang’s model [19,20] is expressed by the following equation:

(7) $$E=E_{0}\;\exp\left[-\left(bp+cp^{2}\right)\right]$$

where p is the porosity; E and $E_{0}$ represent the effective Young’s modulus of porous material and the zero-porosity Young’s modulus, respectively; b and c are the nonnegative material constants. In our study, b and c were set to 1.46 and 9.82, respectively, based on Wang’s studies [19,20] and Spriggs’ study [21]. This model can effectively address the transition from connected to disconnected pores of the same size.

(6) Differential Effective Medium Approach (DEMA):

Pal [5] proposed four models (Table 1) within the Differential Effective Medium Approach (DEMA) to determine the elastic properties of two-phase pore–solid composites. The first two models use porosity as the sole variable, while the other two include the maximum packing volume fraction of pores. This approach creates a concentrated pore–solid composite by gradually introducing pores into a solid matrix until the desired volume fraction is reached.

$E_{m}$ is the Young’s modulus of the non-porous phase, and E is the effective Young’s modulus. The volume fraction of pores is ∅. Models 1 and 2 are accurate for low to moderate porosity, but fail at high porosity due to the ‘crowding effect’ [5]. Models 3 and 4 include the maximum packing volume fraction ($\emptyset_{m}$). For spheres with a size ratio of 0.45, the maximum packing fraction is 0.684. For mono-sized spheres, it is 0.64 [5,22]. Pal [5] showed that Models 3 and 4 consider pore size distribution’s effect on elastic properties.

### 2.2. Microstructure-Free Finite Element Modeling and Construction of Representative Volume Elements

Microstructure-free finite element modeling (MF-FEM) [10] is a recently developed numerical approach for predicting the effective elastic properties of composite materials reinforced by fine particles using a representative volume element (RVE). A prerequisite of MF-FEM is that the characteristic size of particles is much smaller than that of the RVE, specifically about 1/50 for two-phase particulate composites. Therefore, the porous material included in the RVE can be considered as quasi-homogeneous and quasi-isotropic. With this prerequisite, the RVE’s elastic properties are independent of the size and shape of the particles, which can thus be represented by elements. In this paper, MF-FEM is extended to predict the effective properties of porous materials. A key step of MF-FEM is constructing the RVE. The construction of RVEs for both irregular and regular pore distributions is described briefly below.

**Figure 1:**
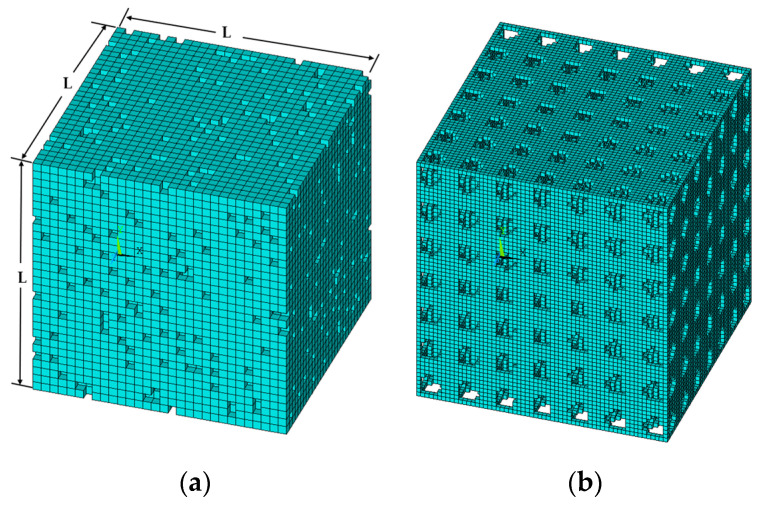
Representative volume element (RVE): (**a**) irregular pore distribution, (**b**) regular pore distribution.

Distinct from other finite element approaches, which first create a geometric model for the RVE with the details of the microstructure and then generate a finite element mesh, MF-FEM first generates a finite element mesh consisting of uniform brick elements. These elements are then assigned intended attributes, such as phase material properties or pores. Sample RVEs of porous material with irregular and regular pore distributions are shown in Figure 1. The RVEs are cubic in shape and have dimensions of L. The empty elements represent pores. It should be noted that a coarse mesh has been used to illustrate the microstructural details; the actual meshes used in the predictions are much finer.

The porosity in the RVEs is calculated as:

(8) $$Porosity\;(\%)=\frac{Total\;number\;of\;the\;pore\;elements}{Total\;number\;of\;the\;RVE\;elements}\times 100$$

The effective elastic properties of the porous materials represented by the RVEs are determined by applying appropriate boundary conditions. The boundary conditions described in Table 2 are applied to the RVEs in Figure 1, where x, y, and z refer to the coordinate system in Figure 1a. $E_{x}$, $E_{y}$, and $E_{z}$ are the effective Young’s modulus in the three axial directions, while $u_{x}$, $u_{y}$, and $u_{z}$ represent the displacements in the three axial directions. Finite element analyses of the RVEs are carried out in ANSYS Mechanical APDL (2021 R2).

After finite element analyses, the effective Young’s modulus of the RVEs are determined by the following equation:

(9) $$E_{i}=\frac{{\bar{\sigma }}_{i}}{{\bar{\epsilon }}_{i}}=\frac{R_{i}∕A_{i}}{\Delta L_{i}∕L_{i}},\;(i=x,\;y,\;z)$$

where ${\bar{\sigma }}_{i}\;and\;{\bar{\epsilon }}_{i}$ denote the nominal stresses and strains, respectively. $A_{i}$ is the area of the surface of RVE (Figure 1); $R_{i}$ is the total reaction force; $L_{i}\;and\;\Delta L_{i}$ are the length and the change in length of RVE side, respectively. If the size of the pores is much smaller than those of the RVE, there will be a very small difference among the Young’s modulus in different axial directions. The following averaging further eliminates these small differences:

(10) $$\bar{E}=\frac{E_{x}+E_{y}+E_{z}}{3}$$

### 2.3. Experimental Cases

Three experimental cases reported in the literature were selected for validation purposes. The properties of the matrix (non-porous) materials in these cases are listed in Table 3. 

The first case reports the effective Young’s modulus of polycrystalline alumina with a porosity up to 40% [21,23]. The second case includes the experimental data of porous ${La}_{0.6}{Sr}_{0.4}{Co}_{0.2}{Fe}_{0.8}0_{3-\delta }\;$ ceramic films sintered at temperatures between 900 °C and 1200 °C, with porosities up to 50% [3,24]. The third case involves a ceramic matrix scaffold material known as CEL2 [25], which achieved high porosity of 77% due to the interconnected voids. The experimental data were either extracted from tabulated data, when available, or extracted from figures in the references using MATLAB functions. Regarding the characteristics of the microstructures of the porous materials in the three cases, the following information can be extracted from the papers. The replication technique in [25] resulted in more regular, strut-like morphologies in the ceramic matrix scaffold, whereas the techniques used in [21,23], and [24] produced more irregular pore distributions in the polycrystalline alumina and ceramic films. 

## 3. Results

**Figure 2:**
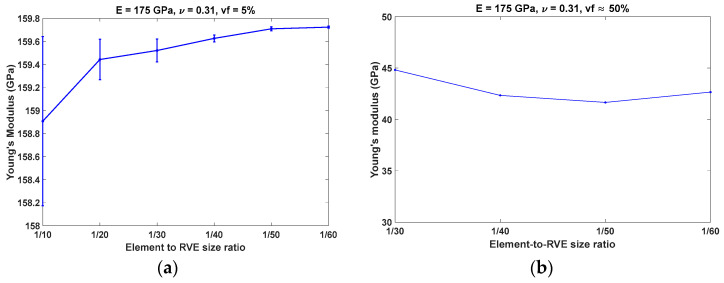
Variation in Young’s modulus with element to RVE size ratio: (**a**) irregular pore distribution; (**b**) regular pore distribution.

The element-to-RVE size ratio for both the regular and random porous structure was determined based on a convergence study, with the results presented in Figure 2. For the irregular pore distribution, a 5% porosity was used in the convergence study, as shown Figure 2a. For regular pore distribution, the porosity was set to approximately 50%, as shown in Figure 2b. Based on the convergence study, an element-to-RVE ratio of 1/60 was selected for both irregular and regular pore distributions. 

**Figure 3:**
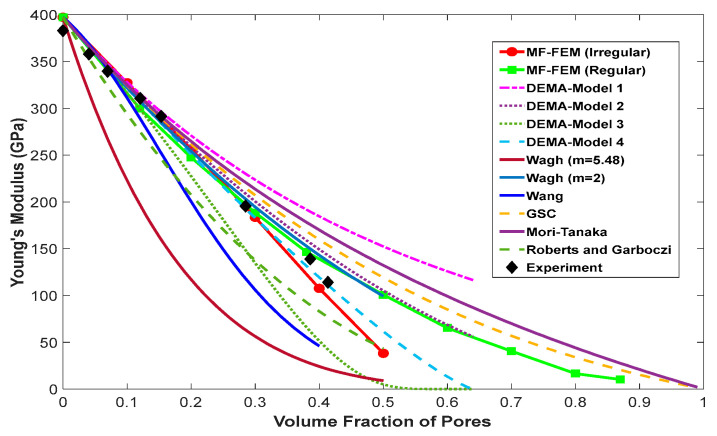
Comparison of the effective Young’s modulus predicted by MF-FEM and micromechanics models with experimental results for porous polycrystalline alumina, as reported in [21,23].

**Figure 4:**
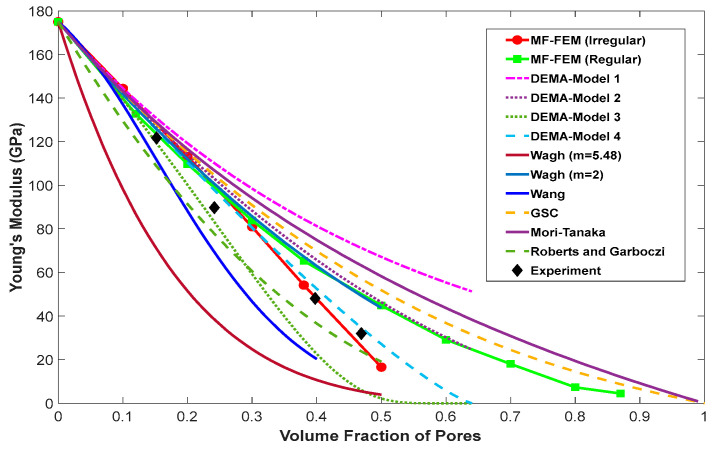
Comparison of the effective Young’s modulus predicted by MF-FEM and micromechanics models with experimental results for porous ceramic films, as reported in [24].

**Figure 5:**
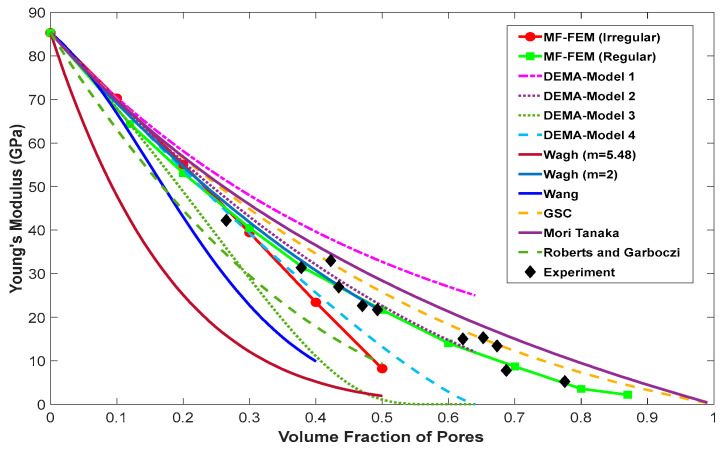
Comparison of the effective Young’s modulus predicted by MF-FEM and micromechanics models with experimental results for porous ceramic films, as reported in [25].

**Figure 6:**
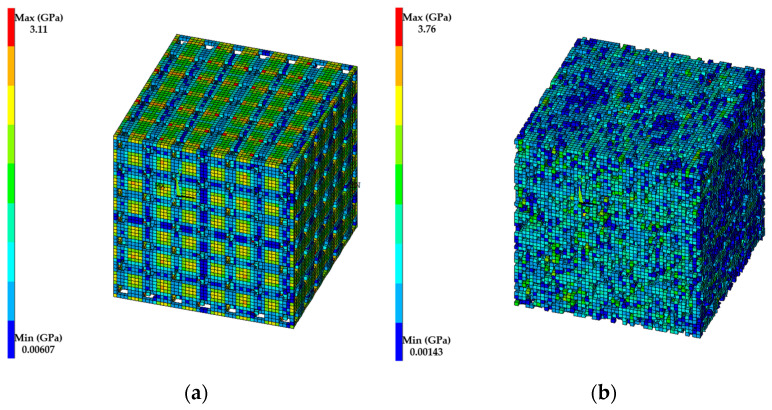
The von Mises stress distribution in RVE with: (**a**) regular pore distribution; (**b**) irregular pore distribution.

Comparisons of the effective Young’s modulus predicted by MF-FEM and the six analytical micromechanics models against the three experimental cases are presented in Figure 3, Figure 4 and Figure 5. The von Mises stress distributions in the RVE with regular and irregular pore distributions, produced using the material properties in Case 2 from Table 3, are shown in Figure 6. 

The following observations can be made from the results presented in Figure 3, Figure 4, Figure 5 and Figure 6:

MF-FEM predictions, whether with regular or irregular pore distributions in the RVE, show excellent agreement with the experimental results. In Figure 3 and Figure 4, MF-FEM predictions with irregular pore distributions align well with the experimental data measured from porous materials with irregular microstructures. In Figure 5, MF-FEM predictions with regular pore distributions demonstrate excellent agreement with the experimental data that have higher regularity in the microstructure.

Even with the same porosity, MF-FEM shows a significant difference in the effective Young’s modulus when pores are regularly versus irregularly distributed, especially at higher porosity levels. With irregular pore distribution, the effective Young’s modulus decreases rapidly at around 50% porosity. In contrast, for regular pore distribution, the decrease in effective Young’s modulus is gradual and diminishes after 90% porosity.

When using MF-FEM predictions as a reference, the performance of micromechanics models varies, especially at higher porosity levels (>40%). For irregular pore distributions, models such as DEMA-Model-4 and the Roberts and Garboczi equation are more accurate. Conversely, for regular pore distributions, models like DEMA-Model-2, Wagh (m = 2), GSC, and the Mori–Tanaka equation provide better accuracy.

The von Mises stress distribution in the RVE with regular pore distribution (Figure 6a) is more uniform than in the RVE with irregular pore distribution (Figure 6b). The maximum von Mises stress observed in the regular pore distribution is 3.11 GPa, while the irregular pore distribution exhibits a higher maximum stress of 3.76 GPa.

## 4. Discussion

The study results show that (1) irregular versus regular pore distribution has a significant effect on the effective Young’s modulus of porous materials, particularly at high porosity levels. The Young’s modulus of porous materials with regular pore distribution is much higher than that of materials with irregular pore distribution. (2) Some micromechanics models are more accurate for porous materials with regular pore distribution, while others are more accurate for irregular pore distribution. The mechanisms behind these two phenomena are explored and discussed below. 

The significant effect of pore distribution on the effective Young’s modulus of porous materials, particularly at high porosity levels, can be attributed to several factors. In materials with regular pore distribution, the uniform spacing and consistent size and shape of the pores allow for even load distribution across the material, reducing stress concentrations and enhancing structural integrity. This regular arrangement creates predictable and uniform load paths, maintaining higher stiffness and strength. Conversely, materials with irregular pore distribution exhibit varying pore sizes and shapes randomly dispersed throughout the material, leading to stress concentrations and localized deformation, which lower overall stiffness. This is evidenced by the patterns of von Mises stresses in Figure 6. Additionally, regular pore structures often result in limited connectivity between pores, preserving the mechanical properties of the solid matrix. Irregular distributions, however, can lead to a network of interconnected voids, facilitating crack propagation and deformation, thus compromising the material’s load-bearing capacity. At high porosity levels, these differences become more pronounced, with regular structures maintaining a stable framework and irregular structures creating large weak zones. Both experimental observations and computational models, such as MF-FEM, support these findings, demonstrating that regular pore distributions result in higher predicted Young’s modulus values compared to irregular distributions, especially at higher porosities.

The performance of micromechanics models varies significantly depending on whether the pore distribution is irregular or regular. For irregular pore distributions, models such as DEMA-Model-4 and the Roberts and Garboczi equation are more accurate. This is because DEMA-Model-4 incrementally adds pores and recalculates material properties, effectively capturing the interactions between randomly distributed pores and the matrix material. The Roberts and Garboczi model, on the other hand, considers the statistical distribution of pore shapes and sizes, using numerical techniques to model the random nature of the pores accurately. Conversely, for regular pore distributions, models like DEMA-Model-2, Wagh (m = 2), the Generalized Self-Consistent model (GSC), and the Mori–Tanaka equation provide better accuracy. DEMA-Model-2 assumes a more homogeneous addition of pores, aligning well with regular distributions. Wagh’s model with an exponent m = 2 is tailored for uniformly distributed pores, offering higher accuracy. The GSC method considers the interaction between a representative inclusion and the surrounding matrix, making it effective for regular distributions. Similarly, the Mori–Tanaka model assumes a homogenous distribution of inclusions, calculating effective properties by considering average stress and strain fields, thus providing accurate predictions for regular pore structures. Therefore, the choice of model should be based on the pore distribution characteristics to ensure the most accurate predictions.

Regular pore distributions offer predictable mechanical properties, uniform stress distribution, and improved fluid permeability, making them ideal for applications like bone scaffolds and filtration systems. However, they are often more complex and costly to manufacture. In contrast, irregular pore distributions are easier and cheaper to produce and are useful in applications like impact absorption, where random stress distribution and energy dissipation are beneficial. The main drawback of irregular pores is their susceptibility to crack propagation due to uneven stress concentrations, which can reduce load-bearing capacity.

## 5. Conclusions

This study aimed to investigate the impact of regular versus irregular pore distributions on the stiffness of porous materials, using MF-FEM validated by experimental results. The findings indicate that pore distribution significantly affects the effective Young’s modulus of porous materials, particularly at higher porosity levels. Regular pore distributions lead to a higher Young’s modulus compared to irregular distributions, due to uniform stress transfer and pore interconnections. Conversely, irregular pore distributions result in lower stiffness, with a rapid decrease in Young’s modulus at porosities exceeding 50%.

The study also evaluated the predictive accuracy of various analytical micromechanics models. It was found that models such as DEMA-Model-4 and the Roberts and Garboczi equation are more accurate for irregular pore distributions, while DEMA-Model-2, Wagh (m = 2), GSC, and the Mori–Tanaka equation better predict the properties of materials with regular pore distributions. These discrepancies are attributed to the underlying assumptions of each model regarding pore shape, size, and distribution. This research underscores the importance of considering pore distribution patterns in modeling to accurately predict the mechanical behavior of porous materials, thus aiding in the design of materials with tailored properties for specific applications.

## Figures and Tables

**Table 1:** Expressions of effective Young’s modulus of the DEMA models [5].

Models	Expression for Young’s Modulus
1	$E=E_{m}\;\exp\left(-\frac{23}{12}\emptyset \right)$
2	$E=E_{m}{\left(1-\emptyset \right)}^{23∕12}$
3	$E=E_{m}\;\exp[\frac{(-23/12)\emptyset }{1-\left(\emptyset ∕\emptyset_{m}\right)}]$
4	$E=E_{m}{\left(1-\frac{\emptyset }{\emptyset_{m}}\right)}^{\left(23/12\right)\emptyset_{m}}$

**Table 2:** Boundary conditions for the analysis of effective properties of materials at the RVE scale [10].

RVE Surface	Young’s Modulus ($E_{i},i=x,y,z$)		
	$E_{x}$	$E_{y}$	$E_{z}$
x = 0	$u_{x}=0$	$u_{x}=0$	$u_{x}=0$
y = 0	$u_{y}=0$	$u_{y}=0$	$u_{y}=0$
z = 0	$u_{z}=0$	$u_{z}=0$	$u_{z}=0$
x = L	$u_{x}=1$	$u_{x}\;(coupled\;DOFs)$	$u_{x}\;(coupled\;DOFs)$
y = L	$u_{y}\;(coupled\;DOFs)$	$u_{y}=1$	$u_{y}\;(coupled\;DOFs)$
z = L	$u_{z}\;(coupled\;DOFs)$	$u_{z}\;(coupled\;DOFs)$	$u_{z}=1$

**Table 3:** Elastic properties of matrix (non-porous) materials and porosity range.

Experiment Case	Young’s Modulus	Poisson’s Ratio	Porosity Range (%)	Reference
1	397.3 GPa	0.25	0–41	[21,23]
2	175.0 GPa	0.31	0–50	[24]
3	85.3 GPa	0.25	0–77	[25]

## Data Availability

The original contributions presented in the study are included in the article; further inquiries can be directed to the corresponding author.
